# Direct-Acting Oral Anticoagulants and Their Reversal Agents—An Update

**DOI:** 10.3390/medicines6040103

**Published:** 2019-10-15

**Authors:** Stephanie A. Kustos, Pius S. Fasinu

**Affiliations:** Department of Pharmaceutical Sciences, College of Pharmacy & Health Sciences, Campbell University, Buies Creek, NC 27506, USA; sakustos0927@email.campbell.edu

**Keywords:** andexanet alfa, anticoagulation, apixaban, betrixaban, dabigatran, direct oral anticoagulants, edoxaban, idarucizumab, rivaroxaban

## Abstract

**Background:** Over the last ten years, a new class of drugs, known as the direct-acting oral anticoagulants (DOACs), have emerged at the forefront of anticoagulation therapy. Like the older generation anticoagulants, DOACs require specific reversal agents in cases of life-threatening bleeding or the need for high-risk surgery. **Methods:** Published literature was searched, and information extracted to provide an update on DOACS and their reversal agents. **Results:** The DOACs include the direct thrombin inhibitor—dabigatran, and the factor Xa inhibitors—rivaroxaban, apixaban, edoxaban, and betrixaban. These DOACs all have a rapid onset of action and each has a predictable therapeutic response requiring no monitoring, unlike the older anticoagulants, such as warfarin. Two reversal agents have been approved within the last five years: idarucizumab for the reversal of dabigatran, and andexanet alfa for the reversal of rivaroxaban and apixaban. Additionally, ciraparantag, a potential “universal” reversal agent, is currently under clinical development. **Conclusions:** A new generation of anticoagulants, the DOACs, and their reversal agents, are gaining prominence in clinical practice, having demonstrated superior efficacy and safety profiles. They are poised to replace traditional anticoagulants including warfarin.

## 1. Introduction

**Figure 1:**
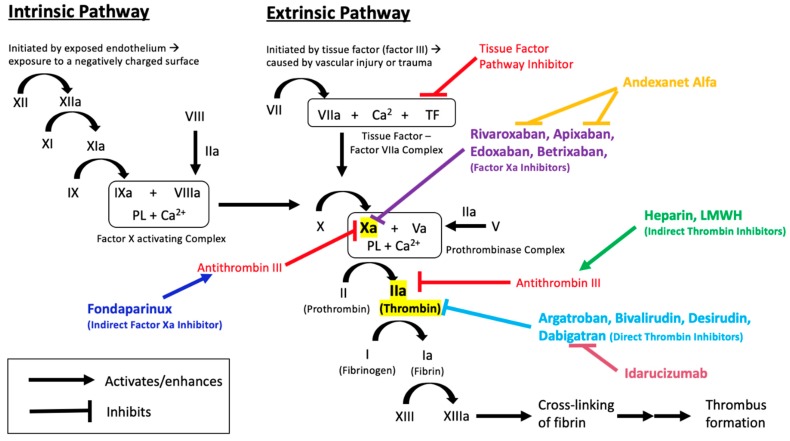
Overview of the coagulation cascade, indicating the sites of action of anticoagulant medications and their reversal agents.

Maintaining the physiologic and therapeutic balance between coagulation and bleeding is necessary for cardiovascular health and sustenance of body functions. This delicate balance is a result of complex physiologic and biochemical processes which, when disrupted, can lead to fatal consequences, such as thrombosis or bleeding [1]. The coagulation cascade, through the interaction of various proteins, clotting factors, and platelets (Figure 1), functions to prevent blood loss in cases of vascular injury.

Anticoagulants are important drugs used as the primary intervention for the prevention and treatment of thrombosis (Table 1). Unfractionated heparin (UFH) and low-molecular weight heparin (LMWH) are often used in acute thrombosis because of their rapid onset of action and effectiveness [2]. UFH and LMWH bind and activate antithrombin, which acts to inhibit factor IIa (thrombin) and factor Xa, inhibiting further progression of the clotting cascade [3]. As a result of this, heparins are considered indirect anticoagulants. Heparins are only bioavailable through parenteral administration, thus excluding the option of easy self-administration. This, in addition to the need to monitor activated partial thromboplastin time (aPTT) (especially with UFH), the risk of heparin-induced thrombocytopenia (HIT), risk of major bleeding episodes, and increased risk of osteoporosis and vertebral fractures, form the major limitations associated with heparin and LMWH therapy. Reversal of these agents is usually not required due to their relatively short half-lives. However, in severe bleeding cases, protamine sulfate is an effective reversal agent for both UFH and LMWH [4]. 

Direct thrombin inhibitors (DTIs), including argatroban, bivalirudin, desirudin, and lepirudin, are US-FDA-approved alternative parenteral anticoagulants. While bleeding is the most common complication associated with DTI use, no specific reversal agent is currently available [5].

Vitamin K antagonists are available for oral administration. They exert pharmacological activity by inhibiting vitamin K epoxide reductase (VKORC1), an enzyme required to convert vitamin K to its reduced and active form. The reduced form of vitamin K acts as a cofactor for gamma-glutamyl carboxylase, an enzyme that is responsible for activating vitamin-K dependent clotting factors (II, VII, IX, X). By inhibiting VKORC1, vitamin K antagonists, such as warfarin, indirectly inhibit the activation of clotting factors II, VII, IX, and X [6]. In addition to the narrow therapeutic index, inter-individual variability in response to warfarin therapy is a major challenge in dosing. Therefore, it is necessary to monitor the international normalized ratio (INR) and adjust the dose of warfarin accordingly. Achieving and maintaining the appropriate INR range is often complicated by external factors, such as diet and drug- or herb-related interactions. Bleeding associated with warfarin is relatively common, and in severe cases, warfarin therapy can be reversed through the administration of vitamin K, fresh frozen plasma (FFP), prothrombin complex concentrate (PCC), or recombinant factor VIIa (rFVIIa) [7].

Recent advancement in drug development has led to the approval of direct-acting oral anticoagulants (DOACs), including direct anti-IIa (dabigatran etexilate) and direct anti-Xa anticoagulants (apixaban, betrixaban, edoxaban, and rivaroxaban). Along with warfarin, the DOACs are now the mainstay of anticoagulant therapy in outpatient settings. DOACs, in particular, have become preferred over warfarin in the prevention and treatment of thromboembolic disorders, primarily because of their proven efficacy, superior safety records, and more predictable and reliable pharmacokinetic and pharmacodynamic profiles [8]. Additionally, the DOACs do not have a requirement for routine coagulation monitoring. DOACs are preferred over VKAs for VTE treatment and prevention, and for stroke prevention in patients with nonvalvular atrial fibrillation (NVAF) [9]. While DOACs have many favorable characteristics, they have limitations. Apart from the cost, bleeding risk is still a major concern. Although there is a believed upward trend in the number of patients reporting bleeding after taking DOACs, several studies, including a systemic review and meta-analysis of existing data, have shown that bleeding rates are actually much lower with DOACs than those associated with VKAs [10,11,12,13].

As a new class of drugs, DOACs require the use of specific reversal agents in cases of life-threatening bleeding or the need for high-risk surgery. Clinicians are still getting acquainted with different approaches to reverse a DOACs’ anticoagulant activity. Therefore, the aim of this study is to provide an updated review on DOACs and their reversal agents based on current literature, guidelines, and recommendations.

## 2. Materials and Methods

This is a non-structured narrative review conducted to provide an overview and update on direct oral anticoagulants and their reversal agents. The review was conducted by searching PubMed, Medline, Cochrane, Web of knowledge, Google Scholar, and Clinicaltrials.gov databases for original studies, case reports, clinical guidelines, and clinical trial reports on DOACs and their reversal agents using relevant search terms and the combinations thereof, including individual anticoagulants, reversal agents, and pharmacological classes. The reference list of retrieved review papers and meta-analyses were also used to identify relevant publications. Inclusion was limited to publications available in the English language.

## 3. Results

Over the last ten years, five DOACs have been FDA-approved, including dabigatran, rivaroxaban, apixaban, edoxaban, and betrixaban. Additionally, two new reversal agents have been FDA-approved within the last five years, specifically for the indication of reversing the effects of some of the DOACs.

### 3.1. Before the Advent of Approved Reversal Agents

**Figure 2:**
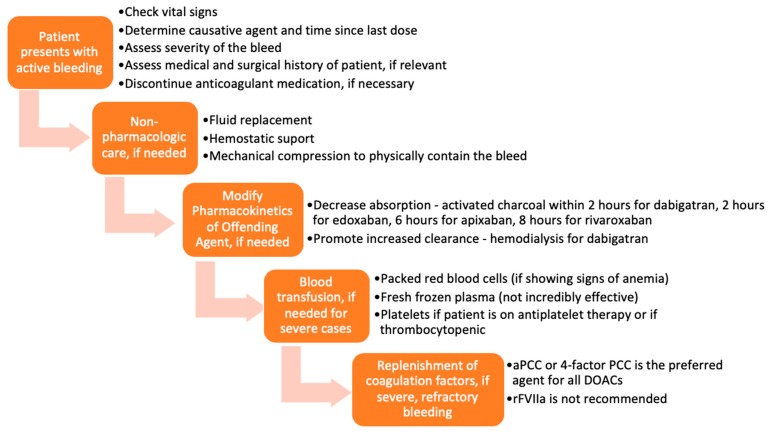
Flow chart of management of bleeding, before the approval of idarucizumab and andexanet alfa.

Various approaches and interventions were utilized to reverse the effects of DOACs before the approval of specific reversal agents. A typical representation of these approaches is presented in Figure 2 [14]. The methods chosen were based on the severity of the DOAC-induced hemorrhage. The success of such measures largely depended on the time the anticoagulant was last administered, the dose administered, concurrent medications, the site of the bleed, and the patient’s co-morbidities. Treatment could be divided into tiers based on the severity of the bleed and the offending agent.

In mild or moderate DOAC-induced bleeding, the standard of care was to delay or withhold subsequent administration of the drug [15]. However, in severe bleeding, more aggressive supportive interventions, including blood transfusions and mechanical compression, were employed [16,17]. Timely administration of activated charcoal (within 2 h of edoxaban [18,19], 6 h of apixaban [20], or 8 h of rivaroxaban [21] use) has been shown to significantly reduce DOAC absorption. Enhancement of clearance through hemodialysis was a particularly useful option for dabigatran, as it is not highly protein-bound and is mainly cleared by the kidneys [22,23]. Hemodialysis has not been shown to be effective in reversing apixaban and rivaroxaban [15,24]. 

Several studies have also examined the use of blood products and coagulation factor replenishment, such as prothrombin complex concentrates (PCC), fresh frozen plasma (FFP), and activated recombinant factor VII (rFVIIa) for the reversal of anticoagulation induced by DOACs [25,26,27,28]. FFP is unlikely to be useful in reversing the DOACs because, as shown in studies, the replenishment of coagulation factors is not sufficient to reverse the direct effects of DOACs on factor IIa and factor Xa [14,29,30,31,32]. PCC is the most widely used and most effective of these blood products for DOAC-related bleeding. More specifically, studies have demonstrated that activated PCC (factor eight inhibitor bypassing activity (FEIBA)) or 4-factor PCC are the preferred agents for DOAC-induced anticoagulation reversal before the approval of specific antidotes [32]. Tranexamic acid has been widely used to manage bleedings—drug-induced or otherwise—with great success [33].

Each of the strategies discussed has a particular place in therapy. Even though specific reversal agents have been developed and are currently under development, non-pharmacologic measures are always important first steps in the therapy for a bleed.

### 3.2. Direct Thrombin Inhibitor–Dabigatran—And Its Reversal

Dabigatran etexilate is currently the only oral direct thrombin inhibitor available. Approved in 2010, dabigatran is indicated for VTE treatment and secondary prophylaxis in patients who have been treated with a parenteral anticoagulant for 5 to 10 days, VTE prophylaxis in total hip arthroplasty (THA), and stroke prevention in patients with atrial fibrillation [34].

Dabigatran etexilate is a pro-drug, and is converted to its active form, dabigatran, by nonspecific esterases in the plasma, gut, and liver, after being rapidly absorbed [35,36,37]. Dabigatran has a quick onset of action of 0.5–2 h with a half-life of about 12 h [38]. The active drug exerts its effects by binding directly to the active site of thrombin [39]. This reversible binding renders thrombin inactive, including both unbound thrombin and fibrin-bound thrombin [40]. The inhibition of thrombin prevents the conversion of fibrinogen to fibrin and ultimately leading to the inhibition of clot formation (Figure 1). 

Dabigatran is only partially metabolized by the liver and undergoes glucuronidation (<10%) [41]. Studies have confirmed this by demonstrating that hepatic impairment only slightly affected the amount of active drug in the body [42]. Dabigatran is primarily eliminated by the kidneys (up to 80%), so renal impairment impacts dabigatran elimination more than hepatic impairment does. Since the elimination of dabigatran is highly dependent on the kidneys, it is not recommended in patients with severe renal impairment (CrCl < 30 mL/min), as accumulation of the drug could occur [43].

The metabolism of dabigatran etexilate is mediated primarily by esterases, while CYP450 enzymes play no relevant role [37]. Therefore, drug interactions due to CYP enzymes are not anticipated. As a substrate for p-glycoprotein, dabigatran is predisposed to drug interactions with p-glycoprotein substrates (i.e., digoxin), inhibitors (i.e., amiodarone, verapamil, ketoconazole) or inducers (i.e., rifampicin, phenytoin, phenobarbital, carbamazepine), which may put patients at risk for thrombosis or severe bleeding [44]. 

Dabigatran is contraindicated in patients with active bleeding or with mechanical prosthetic heart valves [34]. Dose-related adverse reactions have been seen with dabigatran, including gastrointestinal disturbances, reported in 25–40% of patients [45,46]. 

Being a direct thrombin inhibitor, dabigatran causes bleeding with reported incidence of 11–19%, and 2% life-threatening incidences [47]. The long duration of action often complicates the need for emergency surgery, as studies have shown that dabigatran must be discontinued at least 24 h before surgery (or 48 h for procedures associated with a high bleeding risk) for patients with normal renal function. If renal function is compromised, dabigatran should be discontinued even earlier [48]. 

Before the approval of specific antidotes, pharmacologic options, such as recombinant factor VIIa (rFVIIa) or prothrombin complex concentrates (PCC), were considered for life-threatening hemorrhage following dabigatran use [49,50]. However, these agents showed conflicting evidence of efficacy, and were never approved for this indication. 

Idarucizumab (aDabi-Fab), an antibody specific to dabigatran, was developed, and was demonstrated to bind to dabigatran in a way that was structurally similar to dabigatran-thrombin association. Functional clotting assays as well as platelet aggregation studies showed that idarucizumab had no effect on coagulation tests, did not enhance the generation of thrombin, and neither increased nor decreased platelet aggregation [51]. 

Early studies suggested that idarucizumab was safe and capable of reversing dabigatran effects within one minute and maintaining such effect for the duration of the infusion (~25 min) [51,52]. The only limitation was the saturation of the antibody, possibly requiring another dose. A two-part phase I clinical trial of idarucizumab examined its proof-of-concept and determined appropriate dosing [53]. The proof-of-concept study examined the effects of idarucizumab in 47 healthy male volunteers who were randomized to receive varying doses of idarucizumab two hours after the final dose of a 4-day dabigatran pre-treatment. Idarucizumab was found to reverse the effects of dabigatran in a dose-dependent manner, with no serious adverse events reported. Mild adverse events, such as infusion site reactions in a few participants and one episode of epistaxis, were reported in the idarucizumab group. However, there was no difference in the incidence of adverse events between the treatment and placebo groups, indicating tolerability of this reversal agent [53].

In the phase III clinical trial, REVERSE-AD, idarucizumab was efficacious in reversing the anticoagulant effects of dabigatran [54,55,56]. This open-label study included a total of 503 patients divided into two groups: patients with uncontrollable or life-threatening bleeding due to dabigatran and patients who needed to undergo an invasive procedure that required the reversal of dabigatran. The primary end point in this clinical trial was the maximum percentage of reversal of dabigatran anticoagulant effects within four hours of idarucizumab administration. Patients received a total of 5 g of idarucizumab, consisting of two 2.5-g boluses administered less than 15 min apart. Within four hours, the medium maximum reversal was 100%, and in most patients, the reversal was sustained for at least 24 h, independent of renal function, age, or sex. In the bleeding group, 67.7% of the patients had stopped bleeding within 24 h, with the other 32.3% of patients being “undetermined”. In the group of patients about to undergo a procedure, 93.4% were regarded as having normal hemostasis. The mortality rate was ~19% in both groups, and thrombotic adverse events were reported in 4.8% of patients within 30 days, and in 6.8% of patients within 90 days. The relatively high mortality rate and incidence of thrombotic events were attributed to multiple factors, including the age of the participants (median age 78) and their multiple comorbid conditions. Also, all of the thrombotic events that occurred within 72 h after idarucizumab administration were due to failure to re-initiate anticoagulation. During this study, anti-idarucizumab antibodies were also discovered in a small percent of patients (5.6%), with two-thirds of these patients having pre-existing antibodies that were cross-reactive with idarucizumab. This phenomenon needs to be examined further. Overall, this phase III clinical trial was one of the main studies that enhanced the regulatory approval by the USFDA of idarucizumab for dabigatran reversal [57].

Approved in 2015, idarucizumab is effective and specifically indicated for dabigatran-related life-threatening or uncontrollable bleeding as well as for the reversal of dabigatran in the event of an emergency surgery requirement [58,59,60]. The approved dose is two 2.5-g IV bolus infusions administered within 15 min [61].

Apart from being the only approved drug for the reversal of dabigatran effect, idarucizumab’s rapid onset of action and ready-to-use formulation (precluding reconstitution) make it user-friendly in an emergency. However, there are limitations and cautions in idarucizumab use. It is thermolabile and photosensitive, and therefore requires stringent storage conditions. Unopened vials in original packaging are only stable for 48 h at room temperature. Once exposed to light, idarucizumab must be used within six hours [62]. Another drawback of idarucizumab is thrombosis risk. Post-marketing reports described events such as acute ischemic stroke, deep vein thrombosis, pulmonary embolism, myocardial infarction, intracardiac thrombus, right heart failure, and other thromboembolic complications [63]. 

With limited post-approval use data, idarucizumab has shown great effectiveness in the majority of the patient population. However, there have been cases of therapeutic failure with the drug. A case report detailed an unsuccessful reversal of dabigatran by idarucizumab in two different patients who had high concentrations of dabigatran [64]. One patient had an emergency surgery and experienced bleeding, while the other patient simply had severely elevated concentrations of dabigatran. Both were refractory to idarucizumab as well as other non-specific reversal strategies, and both cases ultimately resulted in death. It is important to note, however, that both patients had complex medical conditions that contributed to their hemostasis imbalance. Another example of therapeutic failure was in the case of an 87-year-old patient with severe renal failure who had a rebound of dabigatran levels after idarucizumab, which necessitated additional hemodialysis and subsequent management with heparin [65]. In another case report, life-threatening gastrointestinal bleeding persisted after the administration of idarucizumab, such that additional supportive therapy, including emergent angiography and FEIBA administration, was warranted [66]. 

Overall, both dabigatran and idarucizumab play an important role in the treatment of thromboembolic disorders. While promising data exists, it is still imperative to monitor patients for bleeding events and check for early signs of toxicity in order to ensure the safety and effectiveness of both dabigatran and its specific reversal agent, idarucizumab.

### 3.3. Factor Xa Inhibitors and Their Reversal

The idea of developing an oral factor Xa inhibitor became prominent in the early 2000s, raising the hope of finding an agent for anticoagulation without the many adverse effects and drug interactions associated with warfarin [67]. Early success resulted in the approval of rivaroxaban and apixaban, around the same time as dabigatran [68]. At the time, there was no definitive advantage of using a factor Xa inhibitor over a direct thrombin inhibitor. They were simply different in terms of mechanism and biochemical properties [68]. 

Factor Xa inhibitors are small molecule drugs that bind directly to the active site of factor Xa, rendering it inactive. By inhibiting factor Xa, a key component of the clotting cascade, the rest of the cascade ceases to progress. Bound factor Xa is unable to activate thrombin, ultimately preventing the formation of a clot (Figure 1).

#### 3.3.1. Rivaroxaban

Rivaroxaban was the first factor Xa inhibitor approved by the USFDA. It binds reversibly and competitively to factor Xa with a selectivity that is 10,000 times higher than for other serine proteases without needing a cofactor [69,70]. Rivaroxaban has the ability to bind and inhibit both unbound factor Xa as well as clot-bound factor Xa, ultimately preventing the formation of a thrombus. Multiple clinical trials (RECORD, EINSTEIN and ROCKET AF) evaluated rivaroxaban’s effectiveness at preventing and treating a wide variety of thromboembolic disorders [71,72,73,74,75,76]. The RECORD 1 trials reported a significant reduction in the incidence of total VTE from 3.7% in the enoxaparin group to 1.1% in the rivaroxaban group, with an accompanying reduction in major VTE from 2% to 0.2%, respectively. In the RECORD 2 trial, apart from a reduction in major VTE from 5.1% to 0.6%, rivaroxaban showed 79% relative risk reduction compared to enoxaparin. In the RECORD 3 trial, all efficacy endpoints, primary and secondary, were reduced with total VTE reduction from 18.9% to 9.6% in the rivaroxaban group, compared to the enoxaparin group. In addition, there was 62% relative risk reduction associated with rivaroxaban use compared to enoxaparin. Similar superior effects were demonstrated in RECORD 4 with rivaroxaban showing more effectiveness in reducing total VTE from 10.1% to 6.9%. The EINSTEIN trials compared the use of rivaroxaban in 1731 patients to enoxaparin/VKA in 1718 patients for acute DVT, where rivaroxaban showed non-inferior efficacy with regards to the primary outcomes. In the continued-treatment study, rivaroxaban had superior efficacy. ROCKET AF double-blind trials, conducted among 14,264 patients with NVAF, showed that rivaroxaban was non-inferior to warfarin in preventing stroke or systemic embolism. In 2011, rivaroxaban was approved in the US for VTE prophylaxis and treatment, prevention of stroke in patients with NVAF, and for reduction in the risk of major cardiovascular events in patients with coronary or peripheral artery disease [77].

Rivaroxaban has favorable pharmacokinetic and pharmacodynamics properties [78,79]. It is rapidly absorbed with a time to peak of about 2–4 h, with estimated half-life of 5–9 h. Doses of 15 mg or more should be taken with food, in order to maximize absorption, and dosing frequency is dependent upon the specific indication (either once or twice daily). Metabolism of rivaroxaban is carried out mainly by CYP3A4, while some of the unchanged drug is eliminated via the kidneys, facilitated by p-glycoprotein [80]. Rivaroxaban clearance is reduced in patients with renal impairment [81]. The involvement of both the liver and kidneys in rivaroxaban clearance is expected to decrease the risk for clinically significant drug interactions [82]. The major adverse effect of rivaroxaban is hemorrhage, with GI bleeds being the most common. The incidence of hemorrhage is reported to be 5–28%, and major hemorrhage has been reported in 4% of patients or less [83].

#### 3.3.2. Apixaban

Apixaban is a reversible direct factor Xa inhibitor, inhibiting both free and clot-bound factor Xa with a 30,000-fold selectivity compared to other coagulation proteases. It requires no co-factor and does not significantly impact platelet aggregation [84]. Apixaban demonstrated safety and efficacy in various clinical trials, including the ADVANCE, ARISTOTLE and AVERROES trials [85,86,87,88]. In the ADVANCE-1 randomized double-blind phase III trial, oral 2.5 mg twice-daily apixaban was compared to 30 mg subcutaneous twice-daily enoxaparin for the prophylaxis of VTE after total knee arthroplasty in 3195 patients. Apixaban was non-inferior to enoxaparin in efficacy outcome, but was superior in safety outcomes for major bleeding episodes. In the ADVANCE-2, a multicenter phase 3 trial, oral apixaban 2.5 mg twice-daily was more effective than, and as safe (without increased bleeding) as 40 mg once-daily subcutaneous enoxaparin in thromboprophylaxis after knee replacement surgery. In ADVANCE-3 trials, oral 2.5 mg twice-daily enoxaparin was superior to 40 mg once-daily subcutaneous enoxaparin for the prevention of all VTE, all-cause death, and major VTE in patients who have undergone total hip replacement. In the ARISTOTLE trial, apixaban was superior to warfarin in safety (reduced bleeding episodes) and the reduction of stroke, and systemic embolism in patients with atrial fibrillation. In the AVERROES trial, apixaban demonstrated superiority over aspirin in the prevention of stroke in patients with atrial fibrillation. The USFDA approved apixaban in 2012 for use in reducing the risk of stroke and systemic embolism in patients with NVAF. Its indication was expanded in 2014 by the USFDA to include the prevention of DVT and pulmonary embolism in people that had recently undergone knee or hip replacement.

Apixaban has predictable dose-dependent pharmacokinetics and can be taken without meals [89]. It is eliminated via a multiple route, which deceases its risk for drug-drug interaction [90,91,92].

#### 3.3.3. Edoxaban

Edoxaban was approved in 2015 as the third factor Xa inhibitor. Apart from being as effective and well tolerated as apixaban and rivaroxaban, edoxaban offered the additional benefit of long half-life, allowing for a once-daily administration and the potential for better patient compliance [93]. Initial preclinical and clinical studies showed favorable safety profiles with dose range of 10–150 mg, with no dose-dependent adverse events [94,95,96]. As a substrate of P-glycoprotein and CYP3A4, edoxaban has the risk of drug interaction similar to rivaroxaban and apixaban [97,98]. Edoxaban can be taken without regard to food [99].

In the phase III trial, STARS J-V, edoxaban was superior to enoxaparin for the prevention of VTE in patients undergoing total hip arthroplasty in Japan [100]. In the ENGAGE AF-TIMI trial, a phase III trial of edoxaban in the United States, edoxaban was effective, and non-inferior to warfarin in patients with NVAF, with a significantly less incidence of bleeding and cardiovascular death [101]. The pivotal study that led to the approval of edoxaban was the HOKUSAI VTE trial. In this double-blind multicenter study, 8242 patients with acute VTE, who had been initially treated with heparin, were randomized to receive either edoxaban or warfarin for a period of 3 to 12 months. With respect to the primary efficacy outcome of recurrent symptomatic VTE (3.2% in edoxaban vs. 3.5% in warfarin group), edoxaban was non-inferior to warfarin. With regards to the primary safety outcome measured by major or clinically relevant non-major bleeding, edoxaban was safer, with 8.5% outcome as against 10.3% with warfarin [102]. Hemorrhages, including dermal bleeding, GI bleeds, vaginal bleeding, and epistaxis are reportedly more frequent and severe with edoxaban [103]. Edoxaban has several USFDA boxed warnings, including increased risk of ischemic stroke in patients with NVAF who have a creatinine clearance above 95 mL/min, increased risk of ischemic events with premature discontinuation of edoxaban, and the occurrence of spinal or epidural hematomas in patients undergoing spinal puncture or neuraxial anesthesia.

#### 3.3.4. Betrixaban

Betrixaban is the fourth and newest addition to the growing list of factor Xa inhibitors following its approval by the USFDA in 2017. It has a more favorable pharmacokinetic profile with clearance independent of CYP-mediated metabolism, reducing the risk for drug metabolism [104,105,106]. With an onset of action and peak at around 3–4 h, and a half-life of 19–27 h, betrixaban is typically administered once daily. 

In the phase II EXPERT trial, betrixaban demonstrated rapid and effective factor Xa inhibition with dose-dependent effectiveness in preventing VTE in patients undergoing total knee replacement surgery. Participants received a post-operative twice-daily oral betrixaban (15 or 40 mg) or 30 mg subcutaneous twice-daily enoxaparin for 10 to 14 days. VTE incidence, the primary outcome of efficacy, was 20% in the 15 mg betrixaban group, 15% in the 40 mg betrixaban group, and 10% in the enoxaparin group. No major bleeds were reported in the betrixaban group with 2.3% in the enoxaparin group [107]. In another phase II trial, EXPLORE-Xa, betrixaban was compared to warfarin for stroke prevention in patients with atrial fibrillation [108]. The study reported similar bleeding rates between betrixaban and warfarin. However, the bleeding rates seemed to follow a dose-dependent manner as well, perhaps indicating that higher doses of betrixaban would translate to a higher risk of bleeding. In the phase III trial, APEX, extended duration betrixaban was not more effective than enoxaparin at preventing VTE [109]. 

Like other DOACs, the main adverse effects of betrixaban is bleeding (GI bleeding, intracranial hemorrhage, intraocular hemorrhage, hematuria, and epistaxis), reported in 1–2% of patients [110]. As a relatively new drug, data on the safety of betrixaban in various sub-populations is sparse. However, post-marketing clinical trials are ongoing. These include a current trial aimed to evaluate the pharmacokinetics, pharmacodynamics, and safety of betrixaban in pediatric population [111]. Another study, the BRAVO trial, is aimed to evaluate thrombotic and bleeding events due to betrixaban [112].

#### 3.3.5. Andexanet Alfa

Although all four approved factor Xa inhibitors share the same mechanism of action and are considered safe, bleeding is still a major concern. The variations in their structures are believed to account for the differences in their pharmacokinetics profiles, which also impacts each of their unique indications [113]. In the development of andexanet as a reversal agent for factor Xa inhibitors, consideration was given for the possibility of having a single agent reverse the effect of multiple factor Xa inhibitors. Thus, unlike with dabigatran’s reversal agent, idarucizumab, the use of monoclonal antibodies for reversing factor Xa inhibitors was not a plausible option [114]. 

Andexanet alfa (PRT064445, r-antidote), is an inactivated, recombinant form of human factor Xa and acts as a “decoy”, mimicking the binding of factor Xa inhibitors [115]. Andexanet sequesters the factor Xa inhibitors and renders the drugs incapable of binding and inhibiting factor Xa, thereby reversing any anticoagulant effects [116,117,118]. Fondaparinux and LMWH enhance the action of antithrombin III, which inhibits both thrombin (factor II) and factor Xa. Andexanet competes against factor Xa for fondaparinux-activated or LMWH-activated antithrombin, which would free up more factor Xa to continue to propagate the clotting cascade, thereby partially reversing anticoagulant effects of fondaparinux and LMWH [119]. Additionally, andexanet has the ability to decrease tissue factor pathway inhibitor (TFPI) [120]. TFPI is a key regulator of the clotting cascade, which prevents excessive clotting. By decreasing TFPI, the clotting cascade leans towards thrombus formation. This may lead to thrombotic side effects, a phenomenon which would need to be examined with andexanet. 

In preclinical trials, andexanet restored hemostasis in rat, mouse, and rabbit models [121,122]. In a “first-in-man” dose-ascending pharmacokinetic and pharmacodynamics phase I trial of andexanet in healthy volunteers, which was followed by in vitro anti-factor Xa activity assay, andexanet reversed thrombin generation and the anti-factor Xa activity of rivaroxaban in a dose-dependent manner [123]. A four-module phase II trial in healthy individuals tested the ability of varying doses of andexanet to reverse various anticoagulants; apixaban, rivaroxaban, enoxaparin, and edoxaban [124]. In the apixaban module, andexanet decreased the concentration of free apixaban, reduced anti-factor Xa activity, and re-established the generation of thrombin in a dose-dependent manner [125]. Similar effects were found in studies involving rivaroxaban, enoxaparin, and edoxaban, while andexanet decreased the unbound betrixaban concentrations, reducing anti-fXa (anti-factor Xa) activity, and restoring thrombin generation [126]. 

In two phase III trials, ANNEXA-A and ANNEXA-R, the ability of andexanet to reverse the effects of apixaban and rivaroxaban was examined. Patients in the ANNEXA-A study received 5 mg of apixaban twice daily for 3.5 days, while patients in the ANNEXA-R study received 20 mg rivaroxaban once daily for 4 days. Andexanet was administered 3 h after the last apixaban or placebo dose in the ANNEXA-A study, and andexanet was administered 4 h after the last rivaroxaban or placebo dose in the ANNEXA-R study. The primary outcome in both studies was the percent change in anti-factor-Xa activity from baseline (before administration of andexanet or placebo) to nadir (2 to 5 min after administration of andexanet or placebo). Secondary outcomes included number of participants with >80% reduction in anti-fXa activity from baseline to nadir, change in free factor Xa inhibitor concentration from baseline to nadir, change in thrombin generation, and number of participants with thrombin generation above the lower limit of normal range. Participants included men and women between the age of 50 and 75 years, but excluded anyone who had a history of abnormal bleeding or thrombosis, active bleeding, adult asthma, use of inhaled medications, and those with risk factors for bleeding or thrombosis. Andexanet was found to reverse the anticoagulant effects of apixaban and rivaroxaban within two minutes of administration, an effect that was maintained for the duration of the infusion [120,127]. In the apixaban study, participants who received andexanet experienced a 94% reduction in anti-fXa activity, compared to 21% who received placebo. In the rivaroxaban study, participants who received andexanet experienced a 92% reduction in anti-fXa activity, compared to 18% who received placebo. Furthermore, both studies reported significant decrease in the concentration of unbound apixaban or rivaroxaban and both studies saw restoration of thrombin generation that had been inhibited by the factor Xa inhibitors. All participants who received the full dose of andexanet had >80% reduction in anti-Xa activity. No significant adverse effects, including thrombosis, were reported. No antibodies against factor X or Xa were detected in the trial participants [127]. There was a slight increase in D-dimer and prothrombin fragments (F1 + 2) following andexanet administration, but the levels normalized within 24–72 h. This could be explained by the inhibition of TFPI by andexanet, which would encourage clot formation. However, because these two markers are somewhat non-specific, an increase in levels does not always indicate pro-thrombosis. 

Following the ANNEXA-A and ANNEXA-R studies, a phase III/IV trial, the ANNEXA-4 study, examined the reversal effects of andexanet on apixaban- or rivaroxaban-induced anticoagulation in cases of acute major bleeding [128]. The study evaluated 352 patients who had acute major bleeding within 18 h after rivaroxaban or apixaban administration. The trial had two primary outcomes: percent change in anti-fXa activity and percentage of participants with good or excellent hemostasis 12 h following andexanet infusion. Primary safety outcomes included rate of thrombosis, mortality rate, and antibody development. The results demonstrated that andexanet significantly decreased anti-Xa levels by 92% in both the rivaroxaban and apixaban groups, confirming a significant difference in one of the primary outcomes. The other primary outcome of excellent or good hemostasis was observed in 82% of patients 12 h after the end of the andexanet infusion. The study reported thrombotic events in 10% of the participants and a mortality rate of 14% within 30 days following the administration of andexanet. The high rate of thrombosis and mortality called for concern; however, it is important to note that all the patients included had a medical history significant for cardiovascular disorders, and the average age of the participants was 77 years, both factors that may explain the high thrombosis and mortality rates. This study confirmed the ability of andexanet to reduce anti-fXa activity caused by apixaban and rivaroxaban. This study also went a step further and examined andexanet in cases of acute major bleeding, which ultimately became one of andexanet’s clinical indications. 

Following several of these favorable results, and being the first of its kind, andexanet received accelerated approval from the USFDA in May of 2018 for the specific indications of reversing rivaroxaban- and apixaban-related uncontrollable or life-threatening bleeding [129]. A year later (in April 2019), andexanet was approved in Europe for similar indications [130].

**Figure 3:**
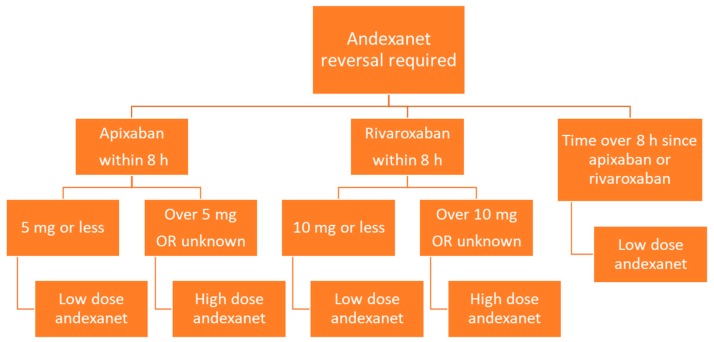
Flow diagram of andexanet dosing based on factor Xa inhibitor, dose, and time since last dose.

Andexanet is administered via intravenous infusion, and dosing depends on the dose of factor Xa inhibitor as well as the time since the factor Xa inhibitor was last administered (Figure 3). Low dose andexanet is 400 mg IV bolus at 30 mg/min, followed by 4 mg/min continuous IV infusion for up to 120 min. High dose andexanet is 800 mg IV bolus at 30 mg/min, followed by 8 mg/min continuous IV infusion for up to 120 min (Table 2).

Andexanet has favorable pharmacokinetic parameters, including a rapid onset of action of 2 min and a short half-life; a pharmacodynamic half-life of about 1 hour and the pharmacokinetic half-life of about 4.5–7.5 h [125,131,132,133]. Elimination pathways are not fully understood at this time. 

The most common adverse effects associated with andexanet use include infusion reactions—with flushing and hot flashes being the most common—and antibody development. The reported antibodies produced were non-neutralizing and not considered to be of serious concern [125]. Some severe adverse effects related to andexanet’s mechanism, reported during clinical trials, include deep vein thrombosis (6%), ischemic stroke (5%), acute myocardial infarction (3%), pulmonary embolism (3%), cardiogenic shock (2%), and cardiac failure (1%) [125]. Other unrelated adverse effects that were reported include pneumonia (≥5%) and urinary tract infections (≥5%). The FDA issued a box warning for the potential for thromboembolic events, ischemic risk, cardiac arrest, and sudden death. Due to the possibility of these serious adverse effects, the patients must be continually monitored. 

While the approval of andexanet has brought about much excitement in the medical community, andexanet is only currently approved for the reversal of the bleeding caused by apixaban and rivaroxaban. This may be because of the lack of evidence to support its use in other factor Xa inhibitors, since edoxaban and betrixaban were very recently approved with limited andexanet-related studies. Regardless, due to its mechanism, andexanet may be able to reverse other anticoagulants as well, and future studies may lead to the expansion of its indications. 

Along with being the only available specific reversal agent for apixaban and rivaroxaban, one other favorable aspect of andexanet is its rapid onset of action, which is desirable for halting active bleeding or achieving prompt reversal prior to an emergency surgery. By contrast, the severe adverse effects, short half-life, mechanism limitations, reconstitution time, and cost associated with andexanet use constitute major limitations in its clinical utility. Andexanet simply binds and sequesters factor Xa inhibitors; it does not induce or accelerate their clearance. This characteristic, along with its short half-life, may necessitate the requirement of multiple doses of the drug if the patient has not yet been stabilized by the time of andexanet’s dissociation and degradation. Andexanet is currently priced at around $3,300 per 100 mg, which would add up to a costly medical treatment of $29,700 for the low dose and $59,400 for the high dose. Another disadvantage is the time-consuming reconstitution process, which may be about 3–5 min per vial. Furthermore, reconstituted vials are stable for only 24 h in the refrigerator or 8 h at room temperature and prepared IV bags with andexanet are stable for only 16 h in the refrigerator or 8 h at room temperature, which prevents batch production of the drug for future use. 

Andexanet is currently undergoing clinical development in Japan, and further clinical utilities are being explored [134]. Another phase II study that is expected to be completed by January 2020 is being conducted to examine the safety, tolerability, pharmacokinetics, and pharmacodynamics of andexanet in a population of healthy Japanese or Caucasian participants exposed to apixaban, rivaroxaban, edoxaban, or placebo [135]. A phase IV trial that started in January 2019 is currently examining the use of andexanet in patients with acute intracranial hemorrhage who are receiving an oral factor Xa inhibitor [136]. Additionally, the ANNEXA-4 extension study is still ongoing to examine the effects of andexanet in patients who have a major bleed while actively taking a factor Xa inhibitor, with the primary outcome being proportion of patients with excellent or good hemostasis 12 h after administration of andexanet [137].

Overall, as the first in its class, andexanet has the potential for expanded indication to reverse all factor Xa inhibitors.

### 3.4. Reversal Agents in Development—Ciraparantag

Ciraparantag, also known as aripazine or PER977, is a small, cationic, synthetic molecule that is being investigated as a potential ‘universal antidote’ to anticoagulants. While the exact pharmacological mechanism of action of ciraparantag is unclear, it is known to bind to various anticoagulant medications through non-covalent bonding, ultimately neutralizing their effects [138,139]. It has been shown to bind to unfractionated heparin, LMWH, fondaparinux, direct thrombin inhibitors, and direct factor Xa inhibitors via charge-charge interactions and hydrogen bonding [140]. Ciraparantag does not appear to interfere with coagulation factors or other proteins involved in the clotting cascade [141].

Preclinical studies of ciraparantag have demonstrated safety in rats and dogs with no toxic effects on the cardiovascular, neurologic, or respiratory systems [142]. A study performed in DOAC-induced bleeding rat tails (using dabigatran, rivaroxaban, apixaban, or edoxaban) found that ciraparantag returned coagulation laboratory values to baseline within 20 min, as well as decreased bleeding [143,144]. In human blood ex vivo, ciraparantag completely reversed both rivaroxaban and apixaban in a dose-dependent manner, measured by anti-Xa activity [145].

A single IV bolus dose (100–300 mg) of ciraparantag in healthy human subjects completely reversed edoxaban-induced anticoagulation within 10 min, which was maintained for 24 h with no major adverse events [140]. A phase I/II trial of ciraparantag examined varying doses (100 mg, 200 mg, or 300 mg) in healthy volunteers treated with 1.5 mg/kg LMWH. Ciraparantag completely reversed the effects of LMWH within 20 min for the 100-mg dose and within 5 min for the 200-mg dose, with no thrombotic event or other serious adverse events reported [141].

While ciraparantag looks to be a promising reversal agent for all of the anticoagulant medications, much is still unknown about its pharmacokinetics and pharmacodynamics, as well as its effectiveness in humans. The FDA has granted fast track designation for the development of ciraparantag, and several clinical trials are underway (Table 3).

## 4. Discussion

DOACs have become a new class of therapeutic agents over the past 10 years, with increasing clinical utility for the prophylaxis and treatment of venous thromboembolism, as well as the prevention of stroke in NVAF. Data on the safety of these agents is sparse, leading many clinicians to be cautious in using them more extensively. Although studies have shown that these agents may present with less bleeding that warfarin, major bleeding is still the primary concern with their use. While warfarin-induced bleeding can easily be halted with the use of vitamin K, the data in reversing DOAC-induced toxicity is limited, even with the relatively new approved reversal agents. 

Warfarin is still widely used despite its many limitations, including the need to monitor INR. DOACs present with several advantages over traditional anticoagulants, including injectables. The predictability of their pharmacological effect and reliable dosing format without the need for monitoring are clinical merits that have made the DOACs preferable to clinicians and patients. Additionally, the non-reliance of DOACs on the CYP pathway for clearance presents an added advantage of reduced risk for drug-drug, food-drug, and herb-drug interactions compared to warfarin. Apart from the cost, the inability to reliably reverse the effect of DOACs has been known as the reason limiting their widespread use. Mild bleeds are less concerning and generally do not require intervention. Severe bleeding is associated with significant morbidity, and even mortality, if not treated immediately and effectively. Off-label use of PCC has been the mainstay of DOAC reversal, in addition to support measures.

Fortunately, extensive research has led to the development of two new antidotes as well as guidelines to direct practitioners should a hemorrhage event occur after the use of DOACs. The reversal agent for dabigatran, idarucizumab, and the reversal agent for rivaroxaban and apixaban, andexanet, brought about much excitement in the medical community. As being the first specific reversal agents for DOACs, these two agents have demonstrated efficacy and reliability, although, post-marketing data is still sparse.

## 5. Conclusions

A new generation of anticoagulants, DOACs, and their reversal agents are gaining prominence in cardiology. Physicians may reliably use DOACs with less concerns about the inability to control associated bleedings, as in the past. Post-marketing data on DOACs, idarucizumab, and andexanet will continue to provide guidance in this field, as DOACs are set to replace warfarin as first-line options in the prevention of venous thromboembolism. It is also exciting to envision the future of a universal reversal agent based on current research trajectory. In all, a lot of progress has been made in this field over the past decade.

## Figures and Tables

**Table 1:** Overview of available anticoagulant medications.

General Class/MOA	Drug Name and Year of First Approval	Labeled Indications	Adult Dosing (with Normal Renal & Hepatic Function)	Route of Administration	Approved Reversal Agent
Vitamin K Antagonist	Warfarin (1954)	VTE prophylaxis and treatment (associated with Afib or cardiac valve replacement) Adjunct to reduce the risk of systemic embolism after MI	INR-adjusted-based dosing Goal INR is 2–3 for most patients Goal INR for mitral valve replacement is 2.5–3.5	Oral	Vitamin K and/or Prothrombin Complex Concentrate
Indirect Thrombin Inhibitors	Heparin (1940s) *	VTE prophylaxis and treatment (associated with thromboembolic disorders or Afib) Prevention of clotting in arterial or cardiac surgery Anticoagulant for extracorporeal circulation or dialysis procedures	VTE Treatment: 80 unit/kg IV bolus, then 18 unit/kg/h IV infusion VTE Prophylaxis: 5000 units q8h Target anti-Xa level 6 h post-dose: 0.3–0.7 units/mL	Injectable Intravenous or Subcutaneous	Protamine → 100% reversal
	Low Molecular Weight Heparins (LMWH): Dalteparin (1994) Enoxaparin (1993) Tinzaparin (2000)	VTE prophylaxis (in hip, knee, abdominal, thoracic, cardiac, or neuro surgery; in patients with restricted mobility; trauma; pregnancy) Thrombosis treatment and secondary prophylaxis (wide variety of indications) Thromboprophylaxis in acute coronary syndrome (unstable angina, NSTEMI, STEMI) or cardioversion in Afib/atrial flutter	DVT Treatment: 1 mg/kg q12h OR 1.5 mg/kg q24h VTE Prophylaxis: 40 mg q24h Target anti-Xa level 4 h post-dose: 0.5–1.1 units/mL	Injectable Subcutaneous	Protamine → 60% reversal
Direct thrombin Inhibitors	Argatroban (2000)	Prophylaxis or treatment of thrombosis in patients with HIT Anticoagulant for percutaneous coronary intervention (PCI)	Prophylaxis/treatment of thrombosis in HIT: 2 mcg/kg/min and adjust based on aPTT (goal 1.5–3 times baseline) PCI: 350 mcg/kg bolus, then 25 mcg/kg/min infusion and adjust based on ACT	Injectable Intravenous	N/A
	Bivalirudin (2000)	Anticoagulant for percutaneous coronary intervention (PCI), including patients with HIT	Before PCI: 0.1 mg/kg bolus, then 0.25 mg/kg/h until PCI During PCI: 0.75 mg/kg bolus, then 1.75 mg/kg/h for the duration of procedure	Injectable Intravenous	N/A
	Dabigatran (2010)	VTE treatment & prevention in patients who have been treated with a parenteral anticoagulant for 5 to 10 days VTE prophylaxis in total hip arthroplasty (THA) Stroke prevention in Afib	VTE Treatment (after initial therapy with a parenteral anticoagulant for 5 days): 150 mg BID Afib: 150 mg BID THA: 110 mg given 1–4 h after surgery, then 220 mg QD for 10–14 d	Oral	Idarucizumab (Praxbind)
	Desirudin (2003)	DVT prophylaxis in hip-replacement surgery	15 mg q12h	Injectable Subcutaneous	N/A
	Lepirudin ** (1998)	HIT Prevention of VTE in patients with HIT	0.4 mg/kg IV bolus, followed by 0.15 mg/kg/h IV infusion for 2–10 days (or as clinically indicated)	Injectable Subcutaneous	N/A
Indirect Factor Xa Inhibitor	Pentasaccharide-Fondaparinux (2001)	DVT or PE treatment in conjunction with warfarin VTE prophylaxis in surgical patients	VTE Treatment: <50 kg → 5 mg QD 50–100 kg → 7.5 mg QD >100 mg → 10 mg QD VTE Prophylaxis: 2.5 mg QD	Injectable Subcutaneous	N/A
Factor Xa Inhibitors	Apixaban (2012)	Treatment of VTE and to reduce recurrence of VTE following initial therapy Prevention of stroke and systemic embolism in NVAF Prophylaxis of VTE post-op in hip or knee arthroplasty	VTE Treatment: 10 mg BID for 7 days, then 5 mg BID Afib: 5 mg BID; if patient has any 2 of the following then 2.5 mg BID → age ≥80, weight ≤60 kg, or SCr ≥1.5 mg/dL Knee/hip arthroplasty: 2.5 mg BID starting 12–24 h after surgery Secondary prevention: 2.5 mg BID (following 6 months of initial therapy)	Oral Tablet	Andexanet Alfa
	Betrixaban (2017)	Prophylaxis of VTE in medical patients	VTE prophylaxis: 160 mg as a single dose on day 1, followed by 80 mg once daily for 35 to 42 days	Oral Capsule	N/A
	Edoxaban (2015)	Treatment and prevention of recurrent VTE following 5–10 days of parenteral anticoagulant Prevention of stroke and systemic embolism in NVAF	VTE: If >60 kg → 60 mg QD If ≤60 kg → 30 mg QD Afib: 60 mg QD	Oral Tablet	N/A
	Rivaroxaban (2011)	Treatment of VTE Prophylaxis of VTE in total hip or knee arthroplasty Prevention of stroke and systemic embolism in NVAF Reduce the risk of cardiovascular events in CAD or PAD Indefinite anticoagulation to reduce the risk of recurrent VTE	VTE treatment: 15 mg BID w/ food for 21 days, followed by 20 mg QD w/ food Knee/hip arthroplasty: 10 mg QD starting 6–10 h after surgery Afib: 20 mg QD w/ evening meal CAD/PAD: 2.5 mg BID (with or without aspirin) Secondary prevention: 10 mg QD (following 6 months of initial treatment) Indefinite anticoagulation: reduced intensity dosing	Oral Tablet	Andexanet Alfa

* Heparin was first described as being an effective anticoagulant as early as 1916, and was grandfathered in after the establishment of the USFDA. ** Bayer, the pharmaceutical company that produced lepirudin, stopped further production of the drug since May 31, 2012. Abbreviations: VTE = venous thromboembolism; Afib = atrial fibrillation; MI = myocardial infarction; DVT = deep vein thrombosis; PE = pulmonary embolism; STEMI = ST-elevated myocardial infarction; NSTEMI = non-ST-elevated myocardial infarction; HIT = heparin-induced thrombocytopenia; CAD = coronary artery disease; PAD = peripheral artery disease Note: VTE includes DVT and PE.

**Table 2:** Dosing of andexanet alfa.

Administration	Low Dose	High Dose
Bolus	400 mg at 30 mg/min	800 mg at 30 mg/min
Infusion	4 mg/min for 120 mins	8 mg/min for 120 mins
Number of vials	Bolus = 400 mg → 4 vials Infusion = 480 mg → 5 vials Total = 9 vials of 100 mg	Bolus = 800 mg → 8 vials Infusion = 960 mg → 10 vials Total = 18 vials of 100 mg OR 9 vials of 200 mg

**Table 3:** Clinical trials studying ciraparantag.

Clinical Trials Identifier	Title	Primary and Secondary Outcomes	Status	Results Available?
NCT01826266	Phase I Evaluation of the Safety, Tolerability, PK, and PD Effects of a Double-Blind, Single Dose of PER977 Administered Alone, and Following a Single Dose of Edoxaban	1º—safety, tolerability, plasma PK, and urinary PK of a range of single IV doses of PER977	Completed December 2013	No
NCT02206100	Phase I/II Evaluation of the Safety, Tolerability, PK, and PD Effects of Single, Hourly-Repeating Escalating Doses of PER977 Following a Single Subcutaneous Dose of Enoxaparin	1º—number of adverse events 2º—reversal of enoxaparin anticoagulation, PK of enoxaparin, PK of PER977 and its metabolite	Completed April 2014	No
NCT02205905	An Open-label, Single-dose, Non-randomized Study of the Absorption, Metabolism, and Excretion of Intravenously Administered 14C-labeled PER977 in Healthy Male Subjects (Phase I Study)	1º—characterization of the mass balance, metabolic disposition, and identification of the metabolites and general metabolic pathways of PER977 2º—number of subjects with adverse events	Completed August 2014	No
NCT02207257	Phase II Randomized, Sequential Group, Evaluation of Ascending Reversal Doses of PER977 Administered to Subjects with Steady State Edoxaban Dosing and Re-anticoagulation With Edoxaban Following PER977 Reversal	1º—WBCT as a measure of edoxaban anticoagulation reversal by PER977 2º—PK of PER977, PK of edoxaban administered with PER977, safety coagulation measures, and safety and tolerability of PER977 after edoxaban	Completed November 2015	No
NCT02206087	Phase I/II Evaluation of the Safety, Tolerability, PK, and PD Effects of a Single Escalating Dose of PER977 Following Administration of Unfractionated Heparin	1º—effect of PER977 on reversal of UFH anticoagulation 2º—safety and tolerability of PER977 administered after UFH	Completed March 2016	No
NCT03288454	Phase 2 Placebo-Controlled, Single-Site, Single-Blind Study of Apixaban Reversal by Ciraparantag as Measured by WBCT	1º—efficacy of ciraparantag in reversal of apixaban-induced anticoagulation, measured by WBCT 2º—number of participants with abnormal lab values or adverse events	Recruiting; expected to be complete in January 2019	No
NCT03172910	Phase 2 Placebo-Controlled, Single-Site, Single-Blind Study of Rivaroxaban Reversal by Ciraparantag as Measured by WBCT	1º—efficacy of ciraparantag in reversal of rivaroxaban-induced anticoagulation, measured by WBCT 2º—incidence of adverse effects	Recruiting; expected to be complete in January 2019	No
